# Adenocarcinoma of the bladder following nephrogenic adenoma: a case report

**DOI:** 10.1186/1752-1947-2-164

**Published:** 2008-05-18

**Authors:** Edwin Hungerhuber, Ekkehard Bach, Arndt Hartmann, Dominic Frimberger, Christian Stief, Dirk Zaak

**Affiliations:** 1 Department of Urology, Klinikum Muehldorf, Krankenhausstrasse, D-84453 Mühldorf a. Inn, Germany; 2 Outpatient Clinic, Moosburg, Biberach, Baden-Wurttemberg, Germany; 3 Department of Pathology, University of Regensburg, Bavaria, Germany; 4 Department of Urology, University of Oklahoma, USA; 5 Department of Urology, Klinikum Grosshadern, University of Munich, Germany

## Abstract

**Introduction:**

Nephrogenic adenomas are generally considered to be benign lesions, but there remains a risk for malignant transformation. Patients with immunosuppression in particular appear to be at a higher risk of malignant disease. We report a case of post-traumatic nephrogenic adenoma in a young patient without immunosuppression, which transformed into an adenocarcinoma of the bladder.

**Case presentation:**

A 25-year-old man had a traumatic bladder perforation caused by a car accident. After physical recovery from the accident, he developed a neurogenic bladder and recurrent urinary tract infections. He presented with nephrogenic adenoma of the bladder 18 months after the accident. The adenoma was treated repeatedly with transurethral resections. The initial pathologic findings were benign, however, the last resection revealed that the former benign adenoma had transformed into a moderately differentiated adenocarcinoma of the bladder (tumor present but no invasion, multifocal, no lymph nodes involved, no metastasis, grade 2). He subsequently underwent radical cystectomy and has remained tumor-free for the last 4 years.

**Conclusion:**

Nephrogenic adenoma is a rare disease with some potential for malignant transformation. However, patients with nephrogenic adenoma under immunosuppression and patients with neurogenic bladder dysfunction appear to be at a higher risk of developing bladder cancer.

## Introduction

Nephrogenic adenoma is a rare tumor which usually occurs in the urinary tract, most frequently in the bladder. Its etiology is unknown, but association with trauma, nephrolithiasis, urinary tract infection and radiation has been reported [1]. Diagnosis is based on histopathologic examination. The therapy of choice in small tumors is transurethral resection, however, a high recurrence rate of 37% to 88% has been reported [2]. Occasionally, these rare tumors are associated with urothelial neoplasms, adenocarcinoma or squamous cell carcinoma of the bladder [2-4].

## Case presentation

A 25-year-old man had a car accident with severe head injury, pelvic fractures, rupture of the symphysis, partial urethral rupture and traumatic bladder perforation at the bladder dome with extension to the right bladder wall. The bladder perforation was treated with primary bladder closure, while the partial urethral rupture was treated with urethral catheterization. The patient's overall health status improved progressively, however, he developed neurogenic bladder dysfunction. Thus, a suprapubic catheter for continuous bladder drainage was necessary for 1 year. One and a half years after suprapubic catheter removal, he presented with sterile microhematuria confirmed by repeated urine examinations. Cystoscopy detected a papillary tumor at the right and posterior bladder wall with extension to the bladder base. The bladder dome was tumor-free. The tumor was removed by transurethral resection (TUR). The histologic diagnosis of nephrogenic adenoma was confirmed by two independent pathologists.

**Figure 1:**
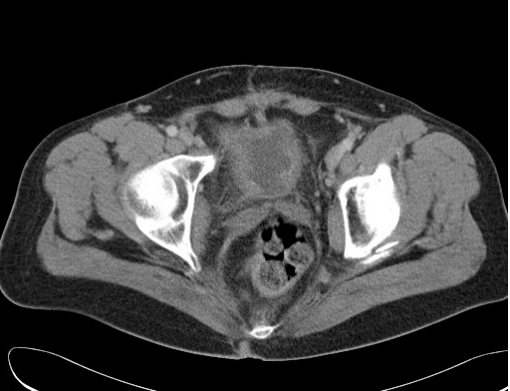
A computed tomography scan showing a thickening of the entire bladder wall with no predominant location, such as the bladder dome.

**Figure 2:**
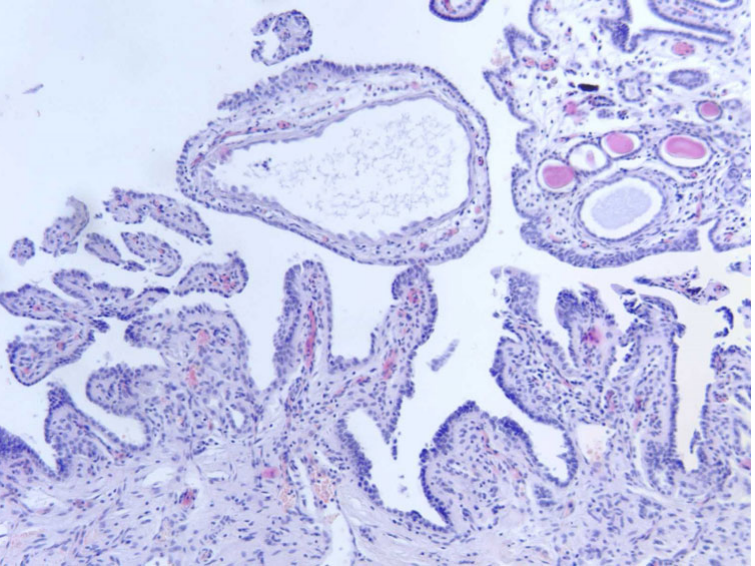
**Histology of a nephrogenic adenoma**. Papillary and tubular growth pattern of nephrogenic adenoma is shown, partly with small cysts with eosinophilic material. This histological picture was found throughout the entire urothelium of the cystectomy specimens (approximately 50% of the urothelial surface of the bladder). Hematoxylin and eosin stain, magnification ×100.

**Figure 3:**
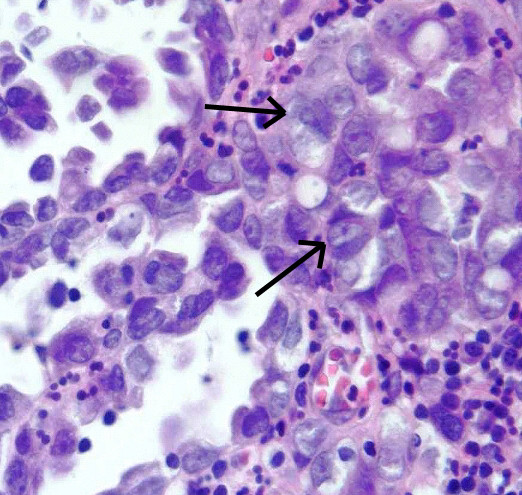
**Histology of a nephrogenic adenoma with focal development of an invasive adenocarcinoma**. Invasive mesonephroid adenocarcinoma of the bladder adjacent to the nephrogenic adenoma is indicated by the arrow. In the right upper quadrant of the histological picture, the focal transformation to mesonephroid adenocarcinoma is shown. Hematoxylin and eosin stain, magnification ×400.

Follow-up consisted of routine urine analysis, cystoscopy and cytology every 3 months. A second TUR was performed because of histologically proven recurrence. The upper urinary tract never showed any signs of tumor spread. Microhematuria occurred again 1 year after initial presentation of the tumor. Cystoscopy now found a diffuse and progressive growing exophytic tumor with extension almost throughout the entire bladder wall (Figure 1). Cytology revealed malignant cells suspicious for adenocarcinoma. Further histologic examination confirmed the reappearance of the nephrogenic adenoma (Figure 2). However, additional focal transformation to a moderately differentiated adenocarcinoma was detected (Figure 3). Staging procedures were negative for metastatic spread. A primary tumor origin in the gastrointestinal system was excluded. A radical cystoprostatectomy with neobladder reconstruction was performed because of the diffuse and extensive growth pattern. Final histology confirmed nephrogenic adenoma with multifocal transformation to a moderately differentiated adenocarcinoma with tubular growth pattern. Postoperative tumor classification was tumor present but no invasion (pT1), multifocal, no lymph nodes involved (pN0), no metastasis M0, grade 2 (G2) and resection shows no tumor (R0). The current follow-up is 4 years and the patient remains tumor-free.

## Discussion

Nephrogenic adenoma was described in 1949 as hamartoma of the bladder [5]; the term nephrogenic adenoma was introduced by Friedman and Kuhlenbeck [6]. Currently this disease is assumed to be a metaplastic process, thus terms such as nephrogenic metaplasia or adenomatoid metaplasia have been established.

Nephrogenic adenoma is a rare tumor, which usually occurs in the urinary tract, most frequently in the bladder. Its etiology is unknown, but association with trauma, nephrolithiasis, infection and radiation has been reported [1]. Most patients are adults with an increased incidence in renal transplant patients [7,8]. In these patients, the renal origin was detected by genetic analysis [9]. About 55% occur in a papillary growth pattern, 35% are sessile and 10% are polypoid. Although most nephrogenic adenomas are small, 10% are larger than 4 cm in diameter [1]. Diagnosis is based on histopathologic examination. The therapy of choice in small tumors is transurethral resection, however, a high recurrence rate of 37% to 88% has been reported [2].

Although nephrogenic adenomas are considered to be benign lesions [5,6], malignant transformations have been reported suggesting a premalignant disease [10], particularly in immunocompromised patients. The malignant entity of nephrogenic adenoma is supposed to be the so-called mesonephroid adenocarcinoma of the bladder [11], but an association to transitional cell carcinoma has also been reported [12]. The first mesonephroid adenocarcinoma was reported in 1968 by Dow and Young [13], but only 15 cases with mesonephroid adenocarcinoma have been published so far. Histologically, a tubular growth pattern is pathognomonic for the mesonephroid adenocarcinoma [14]. The current case of an adenocarcinoma with tubular growth pattern supports the hypothesis of transformation to a mesonephroid adenocarcinoma. A study by Hartmann et al. [15] evaluated molecular genetic hybridization in a similar case and suggested clonal evolution of nephrogenic adenoma to clear cell adenocarcinoma. This result supports the hypothesis in the present case. However, the value of DNA profiling is unclear because the detection of tumor entities with low malignant potential, as in the present case, seems to be limited [16].

A recently described entity that may be similar histologically to adenocarcinoma is a fibromyxoid nephrogenic adenoma [17]. The present case is different and cannot be interpreted as fibromyxoid nephrogenic adenoma because there were no fibromyxoid areas in the tumor and also no spindle cell component.

Patients with neurogenic bladder dysfunction, and especially those with continuous urethral or suprapubic catheter drainage, are known to be at an increased risk of developing bladder tumors. The incidence increases 10 years after the initial catheter placement [18]. The transient suprapubic catheterization in the current patient might have played a role in the development of the present nephrogenic adenoma or adenocarcinoma. This is unlikely, however, since the catheter was only in place for 18 months. In addition, the most common type of bladder cancer in these patients is squamous cell carcinoma rather than adenocarcinoma.

Furthermore the primary tumor was at the right and posterior bladder wall and not at the bladder dome, the former cystostomy site. All these findings do not support the catheter as a primary cause for tumor development. However, etiological relevance of chronic catheter irritation in the development of the malignant transformation cannot be ruled out.

It is reported that adenocarcinomas have their origin from the urachus. These tumors usually arise from the bladder dome, the insertion point of the urachus. The location of the primary tumor, however, does not support the hypothesis in this case. The adenocarcinoma rather occurred multifocally within the nephrogenic adenoma.

## Conclusion

Nephrogenic adenoma is a rare benign disease with some potential for malignant transformation. In particular, in patients under immunosuppression and patients with neurogenic bladder dysfunction, the risk of development of bladder cancer might be increased and should be kept in mind in the surveillance of patients with nephrogenic adenoma.

## Competing interests

The authors declare that they have no competing interests.

## Authors' contributions

EH designed this case report and drafted the manuscript. DZ carried out the operation of this patient and helped to draft the manuscript. CS and EB participated in the design of the study and helped to draft the manuscript. AH carried out the pathological examination. DF made the language corrections. All authors read and approved the final manuscript.

## Consent

Written informed consent was obtained from the patient for publication of this case report and any accompanying images. A copy of the written consent is available for review by the Editor-in-Chief of this journal.
